# 5-(3-Chloro­phen­ylsulfan­yl)-1-methyl-3-trifluoro­methyl-1*H*-pyrazole-4-carbaldehyde *O*-[(2-chloro-1,3-thia­zol-5-yl)meth­yl]oxime

**DOI:** 10.1107/S160053681105358X

**Published:** 2011-12-17

**Authors:** Hong Dai, Shuang Li, Kun-Peng Luo, Jian-Xin Fang, Yu-Jun Shi

**Affiliations:** a College of Chemistry and Chemical Engineering, Nantong University, Nantong 226019, People’s Republic of China; b State Key Laboratory and Institute of Elemento-Organic Chemistry, Nankai University, Tianjin 300071, People’s Republic of China

## Abstract

In the title compound, C_16_H_11_Cl_2_F_3_N_4_OS_2_, the benzene ring and the thia­zole ring make dihedral angles of 83.2 (3) and 78.3 (3)°, respectively, with the pyrazole ring. The crystal packing shows S⋯N contacts of 3.309 (2) Å.

## Related literature

For the bioactivity of pyrazole oxime derivatives, see: Takao *et al.* (1994 ▶); Watanabe *et al.* (2001 ▶). For the biological activity of thia­zole derivatives, see: Fahmy & Bekhit (2002 ▶); Sidoova *et al.* (1999 ▶); Zhang *et al.* (2000 ▶).
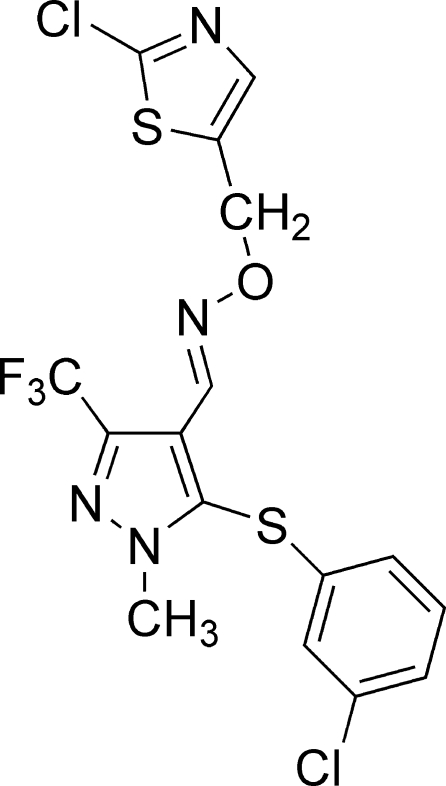


## Experimental

### 

#### Crystal data

C_16_H_11_Cl_2_F_3_N_4_OS_2_
*M* *_r_* = 467.31
Monoclinic, 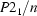

*a* = 12.328 (3) Å
*b* = 12.787 (3) Å
*c* = 13.139 (3) Å
β = 110.16 (3)°
*V* = 1944.3 (9) Å^3^
*Z* = 4
Mo *K*α radiation
μ = 0.59 mm^−1^
*T* = 113 K
0.20 × 0.16 × 0.10 mm

#### Data collection

Rigaku Saturn diffractometer
Absorption correction: multi-scan (*CrystalClear*; Rigaku, 2008 ▶) *T*_min_ = 0.891, *T*_max_ = 0.943
9881 measured reflections
3309 independent reflections
2725 reflections with *I* > 2σ(*I*)
*R*_int_ = 0.116

#### Refinement

*R*[*F* ^2^ > 2σ(*F* ^2^)] = 0.088
*wR*(*F* ^2^) = 0.273
*S* = 1.10
3309 reflections
254 parameters
H-atom parameters constrained
Δρ_max_ = 0.81 e Å^−3^
Δρ_min_ = −0.94 e Å^−3^

### 

Data collection: *CrystalClear* (Rigaku, 2008 ▶); cell refinement: *CrystalClear*; data reduction: *CrystalClear*; program(s) used to solve structure: *SHELXS97* (Sheldrick, 2008 ▶); program(s) used to refine structure: *SHELXL97* (Sheldrick, 2008 ▶); molecular graphics: *SHELXTL* (Sheldrick, 2008 ▶); software used to prepare material for publication: *SHELXTL*.

## Supplementary Material

Crystal structure: contains datablock(s) global, I. DOI: 10.1107/S160053681105358X/aa2035sup1.cif

Structure factors: contains datablock(s) I. DOI: 10.1107/S160053681105358X/aa2035Isup2.hkl

Supplementary material file. DOI: 10.1107/S160053681105358X/aa2035Isup3.cml

Additional supplementary materials:  crystallographic information; 3D view; checkCIF report

## Figures and Tables

**Fig. 1.**
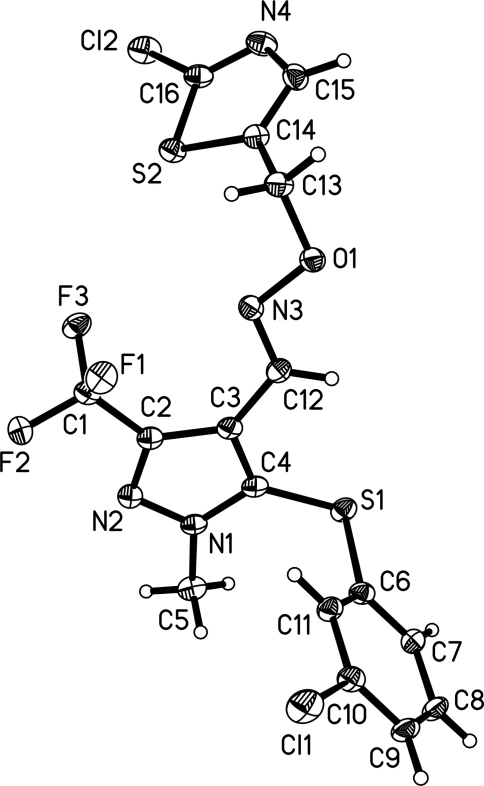
View of the title compound (I), with displacement ellipsoids drawn at the 30% probability level.

## supplementary crystallographic information

### Comment

Recently, pyrazole oximes are reported to possess diverse biological activities, such as fungicidal, insecticidal, and acaricidal activities (Takao *et al.*, 1994; Watanabe *et al.*, 2001). On the other hand, many thiazole derivatives have been found to show insecticidal, herbicidal, and anticancer activities (Sidoova *et al.*, 1999; Zhang *et al.*, 2000; Fahmy & Bekhit, 2002). In search of novel pyrazole oxime derivatives with good bioactivities, we have sought to synthesize new pyrazole oxime ethers containing thiazole units. We report here the crystal structure of the target compound, (I). It contains three planes, the pyrazole ring (C2/C3/C4/N1/N2), the substituted phenyl ring (C6/C7/C8/C9/C10/C11) and the thiazole ring (C15/C14/S2/C16/N4) (Fig. 1). The dihedral angles between the the phenyl ring and the pyrazole ring and between the thiazole ring and the pyrazole ring are 83.2 (3)° and 78.3 (3)°, respectively. The crystal structure of (I) is stabilized by S···N contacts.

### Experimental

To a violently stirred solution of 1-methyl-3-trifluoromethyl-5- (3-chlorophenylthio)-1*H*-pyrazole-4-carbaldehyde oxime (3 mmol) and potassium carbonate (9 mmol) in 20 ml of anhydrous *N*,*N*-dimethylformamide, was added dropwise a solution of 2-chloro-5-chloromethylthiazole (3.6 mmol) in 10 ml of anhydrous *N*,*N*-dimethylformamide. Then, to the above mixture was added a catalytic amount of caesium chloride at room temperature. The resulting solution was heated to 373 K for 6 h. After cooling to room temperature, the mixture was poured into water (200 ml) and extracted with ethyl acetate (3 × 50 ml). The organic layer was washed with 10% sodium carbonate solution (3 × 30 ml) and dried over anhydrous magnesium sulfate. After removal of the solvent, the residue was separated by column chromatography on silica gel using a mixture of petroleum ether/ethyl acetate to obtain colourless crystals.

### Refinement

Although all H atoms were visible in difference maps, they were placed in geometrically calculated positions and included in the final refinement in the riding-model approximation with C—H distances of 0.93–0.97 ° A, and *U*_iso_(H) = 1.2*U*_eq_(C).

### Crystal data

**Table:**

C_16_H_11_Cl_2_F_3_N_4_OS_2_	*F*(000) = 944
*M**_r_* = 467.31	*D*_x_ = 1.597 Mg m^−^^3^
Monoclinic, *P*2_1_/*n*	Mo *K*α radiation, λ = 0.71073 Å
Hall symbol: -P 2yn	Cell parameters from 5433 reflections
*a* = 12.328 (3) Å	θ = 1.6–27.2°
*b* = 12.787 (3) Å	µ = 0.59 mm^−^^1^
*c* = 13.139 (3) Å	*T* = 113 K
β = 110.16 (3)°	Prism, colourless
*V* = 1944.3 (9) Å^3^	0.20 × 0.16 × 0.10 mm
*Z* = 4	

### Data collection

**Table:**

Rigaku Saturn diffractometer	3309 independent reflections
Radiation source: rotating anode	2725 reflections with *I* > 2σ(*I*)
confocal	*R*_int_ = 0.116
ω scans	θ_max_ = 25.0°, θ_min_ = 2.0°
Absorption correction: multi-scan (*CrystalClear*; Rigaku, 2008)	*h* = −11→14
*T*_min_ = 0.891, *T*_max_ = 0.943	*k* = −15→14
9881 measured reflections	*l* = −15→15

### Refinement

**Table:**

Refinement on *F*^2^	Primary atom site location: structure-invariant direct methods
Least-squares matrix: full	Secondary atom site location: difference Fourier map
*R*[*F*^2^ > 2σ(*F*^2^)] = 0.088	Hydrogen site location: inferred from neighbouring sites
*wR*(*F*^2^) = 0.273	H-atom parameters constrained
*S* = 1.10	*w* = 1/[σ^2^(*F*_o_^2^) + (0.2*P*)^2^] where *P* = (*F*_o_^2^ + 2*F*_c_^2^)/3
3309 reflections	(Δ/σ)_max_ = 0.001
254 parameters	Δρ_max_ = 0.81 e Å^−^^3^
0 restraints	Δρ_min_ = −0.94 e Å^−^^3^

### Special details

**Table:**

Geometry. All s.u.'s (except the s.u. in the dihedral angle between two l.s. planes) are estimated using the full covariance matrix. The cell s.u.'s are taken into account individually in the estimation of s.u.'s in distances, angles and torsion angles; correlations between s.u.'s in cell parameters are only used when they are defined by crystal symmetry. An approximate (isotropic) treatment of cell s.u.'s is used for estimating s.u.'s involving l.s. planes.
Refinement. Refinement of *F*^2^ against ALL reflections. The weighted *R*-factor *wR* and goodness of fit *S* are based on *F*^2^, conventional *R*-factors *R* are based on *F*, with *F* set to zero for negative *F*^2^. The threshold expression of *F*^2^ > σ(*F*^2^) is used only for calculating *R*-factors(gt) *etc*. and is not relevant to the choice of reflections for refinement. *R*-factors based on *F*^2^ are statistically about twice as large as those based on *F*, and *R*- factors based on ALL data will be even larger.

### Fractional atomic coordinates and isotropic or equivalent isotropic displacement parameters (Å^2^)

**Table:**

	*x*	*y*	*z*	*U*_iso_*/*U*_eq_	
Cl1	0.01343 (10)	0.94499 (10)	0.66778 (11)	0.0519 (5)	
Cl2	0.57227 (10)	0.80206 (10)	0.00569 (10)	0.0483 (4)	
S1	0.36532 (9)	1.11017 (8)	0.55348 (9)	0.0348 (4)	
S2	0.38132 (9)	0.80301 (8)	0.09658 (8)	0.0334 (4)	
F1	0.2428 (2)	0.69627 (18)	0.3650 (2)	0.0403 (7)	
F2	0.3861 (2)	0.62954 (18)	0.49260 (18)	0.0396 (7)	
F3	0.4134 (2)	0.70073 (18)	0.3550 (2)	0.0388 (7)	
O1	0.1746 (3)	0.9511 (2)	0.1630 (2)	0.0351 (7)	
N1	0.4428 (3)	0.9119 (3)	0.6202 (2)	0.0280 (7)	
N2	0.4393 (3)	0.8114 (2)	0.5871 (3)	0.0293 (8)	
N3	0.2447 (3)	0.8956 (2)	0.2545 (3)	0.0312 (8)	
N4	0.3842 (3)	0.9182 (3)	−0.0643 (3)	0.0402 (9)	
C1	0.3548 (3)	0.7100 (3)	0.4235 (3)	0.0305 (9)	
C2	0.3752 (3)	0.8122 (3)	0.4828 (3)	0.0283 (9)	
C3	0.3354 (3)	0.9140 (3)	0.4454 (3)	0.0279 (8)	
C4	0.3791 (3)	0.9760 (3)	0.5370 (3)	0.0285 (8)	
C5	0.5030 (4)	0.9391 (3)	0.7338 (3)	0.0381 (10)	
H5A	0.4487	0.9410	0.7714	0.057*	
H5B	0.5386	1.0065	0.7379	0.057*	
H5C	0.5613	0.8876	0.7666	0.057*	
C6	0.2689 (3)	1.1125 (3)	0.6278 (3)	0.0310 (9)	
C7	0.2734 (4)	1.2006 (3)	0.6906 (3)	0.0348 (10)	
H7	0.3280	1.2526	0.6967	0.042*	
C8	0.1934 (4)	1.2099 (3)	0.7451 (4)	0.0405 (11)	
H8	0.1950	1.2690	0.7869	0.049*	
C9	0.1125 (4)	1.1326 (3)	0.7376 (3)	0.0397 (10)	
H9	0.0596	1.1389	0.7734	0.048*	
C10	0.1127 (4)	1.0458 (3)	0.6753 (3)	0.0345 (9)	
C11	0.1877 (3)	1.0346 (3)	0.6185 (3)	0.0328 (9)	
H11	0.1839	0.9763	0.5750	0.039*	
C12	0.2648 (3)	0.9524 (3)	0.3388 (3)	0.0308 (9)	
H12	0.2339	1.0195	0.3321	0.037*	
C13	0.1586 (4)	0.8867 (3)	0.0688 (3)	0.0351 (10)	
H13A	0.0938	0.9134	0.0089	0.042*	
H13B	0.1397	0.8161	0.0841	0.042*	
C14	0.2639 (3)	0.8834 (3)	0.0357 (3)	0.0302 (9)	
C15	0.2824 (4)	0.9380 (3)	−0.0462 (3)	0.0360 (10)	
H15	0.2288	0.9862	−0.0877	0.043*	
C16	0.4424 (4)	0.8484 (3)	0.0046 (3)	0.0355 (10)	

### Atomic displacement parameters (Å^2^)

**Table:**

	*U*^11^	*U*^22^	*U*^33^	*U*^12^	*U*^13^	*U*^23^
Cl1	0.0347 (7)	0.0614 (8)	0.0707 (9)	−0.0063 (5)	0.0322 (6)	−0.0026 (5)
Cl2	0.0299 (7)	0.0728 (9)	0.0460 (7)	0.0030 (5)	0.0179 (5)	−0.0001 (5)
S1	0.0347 (7)	0.0345 (6)	0.0437 (7)	−0.0039 (4)	0.0244 (5)	−0.0030 (4)
S2	0.0270 (7)	0.0436 (7)	0.0312 (7)	0.0031 (4)	0.0119 (5)	0.0028 (4)
F1	0.0242 (13)	0.0447 (14)	0.0503 (15)	−0.0077 (9)	0.0109 (11)	−0.0058 (10)
F2	0.0461 (16)	0.0323 (12)	0.0411 (14)	0.0024 (10)	0.0161 (12)	0.0052 (9)
F3	0.0392 (15)	0.0408 (13)	0.0442 (14)	−0.0027 (10)	0.0244 (12)	−0.0076 (10)
O1	0.0342 (16)	0.0420 (15)	0.0284 (14)	0.0115 (11)	0.0100 (12)	0.0036 (11)
N1	0.0197 (16)	0.0392 (17)	0.0297 (17)	−0.0036 (13)	0.0143 (13)	−0.0025 (13)
N2	0.0219 (17)	0.0339 (17)	0.0336 (18)	−0.0021 (12)	0.0115 (14)	−0.0001 (13)
N3	0.0263 (18)	0.0370 (18)	0.0319 (17)	0.0058 (13)	0.0120 (14)	0.0054 (13)
N4	0.036 (2)	0.054 (2)	0.0329 (19)	−0.0050 (17)	0.0147 (16)	0.0029 (17)
C1	0.022 (2)	0.040 (2)	0.033 (2)	0.0003 (15)	0.0145 (16)	0.0024 (16)
C2	0.0200 (19)	0.037 (2)	0.032 (2)	−0.0028 (14)	0.0142 (16)	−0.0006 (15)
C3	0.0203 (19)	0.0370 (19)	0.032 (2)	−0.0006 (14)	0.0160 (16)	0.0011 (15)
C4	0.0191 (18)	0.040 (2)	0.034 (2)	−0.0026 (15)	0.0190 (16)	−0.0001 (15)
C5	0.031 (2)	0.048 (2)	0.035 (2)	−0.0055 (17)	0.0110 (18)	−0.0068 (18)
C6	0.026 (2)	0.040 (2)	0.0281 (19)	0.0018 (15)	0.0111 (16)	0.0017 (15)
C7	0.036 (2)	0.035 (2)	0.036 (2)	0.0047 (15)	0.016 (2)	0.0016 (15)
C8	0.042 (3)	0.047 (2)	0.036 (2)	0.0157 (19)	0.019 (2)	−0.0039 (18)
C9	0.033 (2)	0.056 (3)	0.035 (2)	0.0158 (19)	0.0181 (19)	0.0011 (19)
C10	0.024 (2)	0.046 (2)	0.036 (2)	0.0040 (16)	0.0134 (17)	0.0046 (17)
C11	0.026 (2)	0.041 (2)	0.033 (2)	0.0066 (16)	0.0125 (17)	0.0007 (16)
C12	0.025 (2)	0.036 (2)	0.035 (2)	0.0039 (15)	0.0158 (17)	0.0010 (15)
C13	0.029 (2)	0.047 (2)	0.029 (2)	0.0040 (16)	0.0098 (17)	0.0009 (16)
C14	0.0233 (19)	0.038 (2)	0.0286 (19)	0.0009 (15)	0.0077 (16)	−0.0017 (15)
C15	0.033 (2)	0.040 (2)	0.034 (2)	0.0066 (16)	0.0105 (18)	0.0056 (16)
C16	0.030 (2)	0.047 (2)	0.031 (2)	−0.0041 (17)	0.0120 (17)	−0.0030 (17)

### Geometric parameters (Å, °)

**Table:**

Cl1—C10	1.756 (4)	C3—C12	1.456 (5)
Cl2—C16	1.703 (4)	C5—H5A	0.9600
S1—C4	1.744 (4)	C5—H5B	0.9600
S1—C6	1.780 (4)	C5—H5C	0.9600
S2—C16	1.729 (4)	C6—C7	1.386 (6)
S2—C14	1.730 (4)	C6—C11	1.387 (6)
F1—C1	1.341 (5)	C7—C8	1.409 (6)
F2—C1	1.338 (5)	C7—H7	0.9300
F3—C1	1.339 (5)	C8—C9	1.383 (7)
O1—N3	1.407 (4)	C8—H8	0.9300
O1—C13	1.442 (5)	C9—C10	1.380 (6)
N1—N2	1.352 (5)	C9—H9	0.9300
N1—C4	1.376 (5)	C10—C11	1.382 (6)
N1—C5	1.461 (5)	C11—H11	0.9300
N2—C2	1.325 (5)	C12—H12	0.9300
N3—C12	1.275 (5)	C13—C14	1.505 (6)
N4—C16	1.298 (6)	C13—H13A	0.9700
N4—C15	1.379 (6)	C13—H13B	0.9700
C1—C2	1.497 (5)	C14—C15	1.366 (6)
C2—C3	1.418 (5)	C15—H15	0.9300
C3—C4	1.386 (5)		
			
C4—S1—C6	101.37 (18)	C6—C7—C8	118.7 (4)
C16—S2—C14	88.5 (2)	C6—C7—H7	120.7
N3—O1—C13	107.9 (3)	C8—C7—H7	120.7
N2—N1—C4	111.4 (3)	C9—C8—C7	121.1 (4)
N2—N1—C5	120.2 (3)	C9—C8—H8	119.4
C4—N1—C5	128.3 (3)	C7—C8—H8	119.4
C2—N2—N1	105.5 (3)	C10—C9—C8	117.9 (4)
C12—N3—O1	109.5 (3)	C10—C9—H9	121.0
C16—N4—C15	108.8 (4)	C8—C9—H9	121.0
F2—C1—F3	107.0 (3)	C9—C10—C11	122.8 (4)
F2—C1—F1	106.7 (3)	C9—C10—Cl1	118.7 (3)
F3—C1—F1	106.9 (3)	C11—C10—Cl1	118.5 (3)
F2—C1—C2	111.1 (3)	C10—C11—C6	118.4 (4)
F3—C1—C2	113.3 (3)	C10—C11—H11	120.8
F1—C1—C2	111.6 (3)	C6—C11—H11	120.8
N2—C2—C3	112.2 (3)	N3—C12—C3	121.1 (4)
N2—C2—C1	117.7 (3)	N3—C12—H12	119.5
C3—C2—C1	130.1 (4)	C3—C12—H12	119.5
C4—C3—C2	103.9 (3)	O1—C13—C14	112.8 (3)
C4—C3—C12	124.5 (4)	O1—C13—H13A	109.0
C2—C3—C12	131.6 (3)	C14—C13—H13A	109.0
N1—C4—C3	107.0 (3)	O1—C13—H13B	109.0
N1—C4—S1	122.7 (3)	C14—C13—H13B	109.0
C3—C4—S1	130.2 (3)	H13A—C13—H13B	107.8
N1—C5—H5A	109.5	C15—C14—C13	127.5 (4)
N1—C5—H5B	109.5	C15—C14—S2	109.1 (3)
H5A—C5—H5B	109.5	C13—C14—S2	123.4 (3)
N1—C5—H5C	109.5	C14—C15—N4	116.8 (4)
H5A—C5—H5C	109.5	C14—C15—H15	121.6
H5B—C5—H5C	109.5	N4—C15—H15	121.6
C7—C6—C11	121.0 (4)	N4—C16—Cl2	122.5 (3)
C7—C6—S1	116.3 (3)	N4—C16—S2	116.7 (3)
C11—C6—S1	122.5 (3)	Cl2—C16—S2	120.8 (3)
			
C4—N1—N2—C2	1.0 (4)	C4—S1—C6—C11	−28.8 (4)
C5—N1—N2—C2	177.4 (3)	C11—C6—C7—C8	0.0 (6)
C13—O1—N3—C12	177.1 (3)	S1—C6—C7—C8	175.6 (3)
N1—N2—C2—C3	0.1 (4)	C6—C7—C8—C9	0.7 (6)
N1—N2—C2—C1	−179.5 (3)	C7—C8—C9—C10	0.2 (6)
F2—C1—C2—N2	13.1 (5)	C8—C9—C10—C11	−1.8 (6)
F3—C1—C2—N2	−107.4 (4)	C8—C9—C10—Cl1	178.2 (3)
F1—C1—C2—N2	131.9 (4)	C9—C10—C11—C6	2.4 (6)
F2—C1—C2—C3	−166.4 (4)	Cl1—C10—C11—C6	−177.6 (3)
F3—C1—C2—C3	73.2 (5)	C7—C6—C11—C10	−1.4 (6)
F1—C1—C2—C3	−47.5 (5)	S1—C6—C11—C10	−176.8 (3)
N2—C2—C3—C4	−1.1 (4)	O1—N3—C12—C3	179.8 (3)
C1—C2—C3—C4	178.4 (4)	C4—C3—C12—N3	167.3 (4)
N2—C2—C3—C12	179.3 (4)	C2—C3—C12—N3	−13.1 (7)
C1—C2—C3—C12	−1.3 (7)	N3—O1—C13—C14	−75.3 (4)
N2—N1—C4—C3	−1.7 (4)	O1—C13—C14—C15	−100.8 (5)
C5—N1—C4—C3	−177.7 (4)	O1—C13—C14—S2	81.4 (4)
N2—N1—C4—S1	179.5 (3)	C16—S2—C14—C15	−1.1 (3)
C5—N1—C4—S1	3.5 (5)	C16—S2—C14—C13	177.0 (4)
C2—C3—C4—N1	1.6 (4)	C13—C14—C15—N4	−177.1 (4)
C12—C3—C4—N1	−178.7 (3)	S2—C14—C15—N4	0.9 (5)
C2—C3—C4—S1	−179.7 (3)	C16—N4—C15—C14	−0.1 (5)
C12—C3—C4—S1	0.0 (6)	C15—N4—C16—Cl2	178.8 (3)
C6—S1—C4—N1	−74.0 (3)	C15—N4—C16—S2	−0.9 (5)
C6—S1—C4—C3	107.5 (4)	C14—S2—C16—N4	1.2 (3)
C4—S1—C6—C7	155.6 (3)	C14—S2—C16—Cl2	−178.5 (3)
